# Antibody Titers After a Third and Fourth SARS-CoV-2 BNT162b2 Vaccine Dose in Older Adults

**DOI:** 10.1001/jamanetworkopen.2022.23090

**Published:** 2022-07-21

**Authors:** Noa Eliakim-Raz, Amos Stemmer, Nassem Ghantous, Asaf Ness, Muhammad Awwad, Yaara Leibovici-Weisman, Salomon M. Stemmer

**Affiliations:** 1 Department of Medicine E, Rabin Medical Center, Beilinson Hospital, Petah Tikva, Israel; 2 Sackler Faculty of Medicine, Tel Aviv University, Tel Aviv, Israel; 3 Department of Oncology, Sheba Medical Center, Tel Hashomer, Israel; 4 Davidoff Center, Rabin Medical Center, Beilinson Hospital, Petah Tikva, Israel

## Abstract

This cohort study evaluates the response to a third and fourth SARS-CoV-2 BNT162b2 vaccine dose among individuals aged 60 years or older by evaluating antispike immunoglobulin G antibody titers before and after each dose.

## Introduction

At the beginning of the fifth SARS-CoV-2 wave in Israel, with the B.1.1.529 (Omicron) variant, the effectiveness of the third SARS-CoV-2 BNT162b2 vaccine (Pfizer-BioNTech) against Omicron was questioned. During the fifth wave, in January 2022, the Israeli Ministry of Health authorized a fourth BNT162b2 dose for individuals aged 60 years or older (a third dose was authorized for such individuals in July 2021, during the fourth SARS-CoV-2 wave).

This study, which is an extension of a prior study,^1^ compared the response to the third and fourth BNT162b2 vaccine doses among individuals aged 60 years or older by evaluating antispike (anti-S) immunoglobulin G (IgG) antibody titers before and after each dose. This population is at high risk of developing severe SARS-CoV-2 disease and was the first to receive authorization for a third and fourth vaccine dose.

## Methods

This study was approved by the ethics committee of the Rabin Medical Center in Tel Aviv, Israel. All participants provided written informed consent. After the authorization of a third SARS-CoV-2 vaccine dose in Israel, the Rabin Medical Center offered this vaccine to employees who worked there, as well as their family members; other participants were recruited at the vaccination center. Exclusion criteria included prior SARS-CoV-2 infection and active malignant neoplasm. Anti-S IgG titers were assessed before (August 4-12, 2021) and 10 to 19 days after (August 16-24, 2021) the third vaccination, as well as before (January 4-20, 2022) and 8 to 17 days after (January 13-30, 2022) the fourth vaccination. Serum samples were immediately transmitted to the microbiological laboratory, where anti-S IgG titers were measured with the SARS-CoV-2 IgG II Quant assay (Abbott Laboratories).^2^ Seropositivity was defined as 50 arbitrary units (AU)/mL or more. All adverse events were recorded as well. Additional information is available in the eAppendix in the Supplement. This report followed the Strengthening the Reporting of Observational Studies in Epidemiology (STROBE) reporting guideline for observational studies.

The difference in anti-S IgG values from before to after the third BNT162b2 vaccine and from before to after the fourth BNT162b2 vaccine were evaluated using the Wilcoxon signed rank test. The Spearman correlation was used to assess the correlation between the anti-S IgG titers and age. Multivariable analysis was performed by fitting a generalized linear model on the log of anti-S IgG antibody values after the fourth dose and included age, days from the first vaccination, and days from the fourth vaccination as continuous variables and sex and comorbid conditions (dyslipidemia, hypertension, obesity, type 1 or 2 diabetes, and ischemic heart disease) as categorical variables (eTable in the Supplement). The χ^2^ test was used to assess statistical difference in SARS-CoV-2 infection rates between patients who were vaccinated 4 times and patients who were vaccinated 3 times.

Statistical analyses are described in the eAppendix in the Supplement. A 2-sided *P* < .05 was considered statistically significant. Statistical analysis was performed using R, version 4.0.2 (R Group for Statistical Computing).^3^

## Results

Ninety-nine participants (60 women [61%]; median age, 70 years [IQR, 66-74 years]) received a third BNT162b2 dose, of whom 57 received a fourth dose (Table). Of these 57 participants, 48 had IgG data before and after the third and the fourth dose and are included in the present analysis. The baseline characteristics of the 48 participants with IgG data before and after the third and the fourth dose were similar to those of the entire cohort. Before the fourth dose, all participants were seropositive. The median IgG titers increased significantly after the third dose (450 AU/mL [IQR, 297-903 AU/mL] before the dose and 27 092 [IQR, 15 423-33 074 AU/mL] a median of 14 days [IQR, 13-16 days] after the dose; *P* < .001) and after the fourth dose (3775 AU/mL [IQR, 2070-7096 AU/mL] before the dose and 28 708 AU/mL [IQR, 18 223-44 710 AU/mL] a median of 11 days [9-13 days] after the dose; *P* < .001) (Table; Figure). No significant correlation was observed between age and IgG titer after the fourth dose (*R* = −0.041; *P* = .78). In a multivariable analysis, none of the evaluated variables was associated with higher IgG titers after the fourth vaccine. No major adverse events were reported. Four of 57 participants (7%) who received 4 vaccine doses (median, 6 days [IQR, 5-7 days] after the fourth vaccine) vs 9 of 42 participants (21%) who received 4 vaccination doses (median, 162 days [IQR, 158-167 days] after the third vaccine) had SARS-CoV-2 infection (*P* = .07). All had asymptomatic to mild infection.

**Table.** Baseline Demographic and Cohort Characteristics Before and After the Third and Fourth BNT162b2 Dose

Characteristic	Participants, No. (%)	
	All (N = 99)	With 4 vaccines (n = 48)
Age, median (IQR), y	70 (66-74)	72 (68-75)
Sex		
Female	60 (61)	28 (58)
Male	39 (39)	20 (42)
Comorbidities		
Dyslipidemia	60 (61)	33 (69)
Hypertension	48 (48)	24 (50)
Obesity	26 (26)	15 (31)
Type 1 and 2 diabetes	19 (19)	8 (17)
Ischemic heart disease	17 (17)	9 (19)
Congestive heart failure	1 (1)	1 (2)
Analysis before third dose		
Seropositivity	96 (97)	46 (96)
Days after first dose, median (IQR)	221 (218- 225)	222 (220-225)
IgG titer, median (IQR), AU/mL	442 (294-966)	450 (297-903)
Analysis after third dose		
Seropositivity	99 (100)	48 (100)
Days after first dose, median (IQR)	236 (232- 240)	238 (234-240)
Days after third dose, median (IQR)	14 (13-17)	14 (13-16)
IgG titer, median (IQR), AU/mL	25 429 (14 538-36 366)	27 092 (15 423-33 074)
Analysis before fourth dose		
Seropositivity	NA	48 (100)
Days after first dose, median (IQR)	NA	378 (374-382)
IgG titer, median (IQR), AU/mL	NA	3775 (2070-7096)
Analysis after fourth dose		
Seropositivity	NA	48 (100)
Days after first dose, median (IQR)	NA	390 (385-393)
Days after fourth dose, median (IQR)	NA	11 (9-13)
IgG titer, median (IQR), AU/mL	NA	28 708 (18 223-44 710)
SARS-CoV-2 infection	13 (13)	3 (6)

Abbreviations: AU, arbitrary unit; IgG, immunoglobulin G; NA, not applicable.

**Figure.**
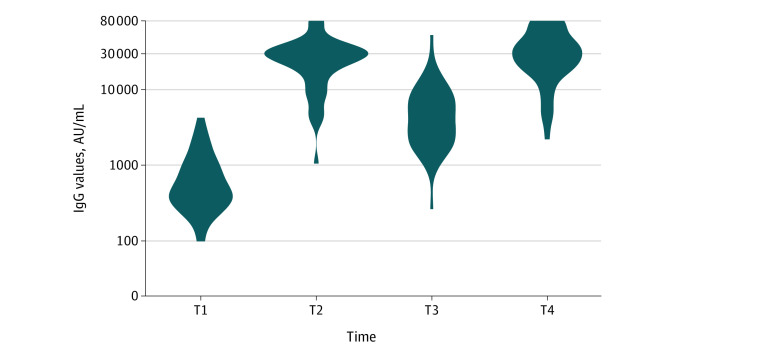
Immunoglobulin G (IgG) Values Before and After the Third and Fourth SARS-CoV-2 BNT162b2 Vaccine Dose T1 indicates the time before the third SARS-CoV-2 BNT162b2 vaccine dose; T2, the time after the third SARS-CoV-2 BNT162b2 vaccine dose; T3, the time before the fourth SARS-CoV-2 BNT162b2 vaccine dose; and T4, the time after the fourth SARS-CoV-2 BNT162b2 vaccine dose.

## Discussion

This study found that the third and fourth BNT162b2 doses in adults aged 60 years or older were associated with a significant increase in anti-S IgG titers approximately 2 weeks after the vaccination, with no major adverse events. Data on the response to the fourth BNT162b2 dose among healthy older adults are lacking. Our findings are consistent with those from case series involving patients who received solid organ transplants, in which significant increases in antibody titers and improved humoral response were associated with the fourth BNT162b2 dose.^4,5^

Study limitations included the small sample size and lack of cellular immunity testing or neutralizing antibody testing, although current evidence suggests that IgG response is a correlate of disease protection.^6^ Also, anti-N IgG titers were not measured; however, all participants were questioned about symptoms and positive test results. The present study did not investigate waning of the immune response after the fourth dose, and therefore it did not address the key question of whether additional boosters would be required.

## Notes

Supplement. **eTable.** Multivariable Analysis of Log IgG Values **eAppendix.** Methods
eReferences.

## References

[1] Eliakim-Raz N, Leibovici-Weisman Y, Stemmer A, . Antibody titers before and after a third dose of the SARS-CoV-2 BNT162b2 vaccine in adults aged ≥60 years. JAMA. 2021;326(21):2203-2204. doi:10.1001/jama.2021.19885 34739043 PMC8652594

[2] Abbott. SARS-CoV-2 immunoassays. Accessed October 1, 2021. https://www.corelaboratory.abbott/int/en/offerings/segments/infectious-disease/sars-cov-2-

[3] R Foundation. The R Project for Statistical Computing. Accessed June 27, 2021. http://www.r-project.org/

[4] Kamar N, Abravanel F, Marion O, . Assessment of 4 doses of SARS-CoV-2 messenger RNA–based vaccine in recipients of a solid organ transplant. JAMA Netw Open. 2021;4(11):e2136030. doi:10.1001/jamanetworkopen.2021.36030 34817587 PMC8613594

[5] Alejo JL, Mitchell J, Chiang TP, . Antibody response to a fourth dose of a SARS-CoV-2 vaccine in solid organ transplant recipients: a case series. Transplantation. 2021;105(12):e280-e281. doi:10.1097/TP.0000000000003934 34428188 PMC8612849

[6] Harvey RA, Rassen JA, Kabelac CA, . Association of SARS-CoV-2 seropositive antibody test with risk of future infection. JAMA Intern Med. 2021;181(5):672-679. doi:10.1001/jamainternmed.2021.0366 33625463 PMC7905701

