# Supplementary material for: Antibody Titers After a Third and Fourth SARS-CoV-2 BNT162b2 Vaccine Dose in Older Adults
Source: JAMA Netw Open. 2022 Jul 21;5(7):e2223090. doi: 10.1001/jamanetworkopen.2022.23090 (PMC9305380; doi:10.1001/jamanetworkopen.2022.23090)
Supplement: Supplement. — eTable. Multivariable Analysis of Log IgG Values eAppendix. Methods eReferences. [file jamanetwopen-e2223090-s001.pdf]

Supplementary Online Content

Eliakim-Raz N, Stemmer A, Ghantous N, et al. Antibody titers after a third and fourth SARS-CoV-2 BNT162b2 vaccine dose in older adults. *JAMA Netw Open.* 2022;5(7):e2223090. doi:10.1001/jamanetworkopen.2022.23090

eTable. Multivariable Analysis of Log IgG Values
eAppendix. Methods
eReferences.

This supplementary material has been provided by the authors to give readers additional information about their work.

© 2022 Eliakim-Raz N et al. *JAMA Network Open.*

eTable. Multivariable Analysis of Log IgG Values

**Characteristic**	**Multivariable analysis**	
	**β (95% CI)**	***P* value**
Age	-0.01 (-0.07 to 0.06)	.87
Sex		
Women	Not applicable	
Men	-0.13 (-0.84 to 0.59)	.73
Days post first vaccination	-0.04 (-0.09 to 0.01)	.15
Days from the fourth vaccination	0.06 (-0.01 to 0.13)	.088
Comorbidities		
Dyslipidemia	-0.25 (-0.90 to 0.39)	.44
Hypertension	0.26 (-0.48 to 1.0)	.50
Obesity	0.12 (-0.46 to 0.69)	.69
Diabetes	-0.03 (-0.8 to 0.73)	.93
Ischemic heart disease	-0.26 (-1.1 to 0.63)	.57

© 2022 Eliakim-Raz N et al. *JAMA Network Open.*

eAppendix. Methods

Anti-S IgG Assessment

Samples (serum) were immediately transmitted to the microbiological laboratory and the titers of IgG antibodies against the SARS-CoV-2 spike receptor-binding domain were determined using a chemiluminescent microparticle immunoassay (CMIA). The assay was performed using the Abbott architect i2000sr platform, in accordance with the manufacturer's package insert for SARS-CoV-2 IgG II Quant assay (Abbott Laboratories, Abbott Park, IL, USA; reference 6S60-22).[1] The strength of the response (in relative light units [RLU]) is determined relative to IgG II calibrator/standard indicates, and reflects the quantity of IgG antibodies present. The assay is 98.1% sensitive ≥15 days after COVID-19 symptoms onset or positive PCR test and 99.6% specific.[2] Seropositivity was defined as ≥50 arbitrary units (AU)/mL.

For all analyses, IgG values above 80,000 AU/mL were considered as 80,000 AU/mL.

© 2022 Eliakim-Raz N et al. *JAMA Network Open.*

eReferences.

1. Abbott. SARS-CoV-2 immunoassay. Accessed October 1, 2021. https://www.corelaboratory.abbott/int/en/offerings/segments/infectious-disease/sars-cov-2-
2. AdviseDx SARS-CoV-2 IgG II. Package insert. Abbott Laboratories; 2021.

© 2022 Eliakim-Raz N et al. *JAMA Network Open.*
